# Additional information on “Direct comparison of the in vitro and in vivo stability of DFO, DFO* and DFOcyclo* for ^89^Zr-immunoPET”

**DOI:** 10.1007/s00259-019-04561-8

**Published:** 2019-11-09

**Authors:** René Raavé, Gerwin Sandker, Pierre Adumeau, Christian Borch Jacobsen, Floriane Mangin, Michel Meyer, Mathieu Moreau, Claire Bernhard, Laurène Da Costa, Adrien Dubois, Victor Goncalves, Magnus Gustafsson, Mark Rijpkema, Otto Boerman, Jean-Claude Chambron, Sandra Heskamp, Franck Denat

**Affiliations:** 1 Department of Radiology and Nuclear Medicine, Radboud Institute for Molecular Life Sciences, Radboud UMC, Nijmegen, The Netherlands; 2 Institut de Chimie Moléculaire de l’Université de Bourgogne, UMR 6302, CNRS, Université Bourgogne Franche-Comté, 9 avenue A. Savary, 21078 Dijon Cedex, France; 3 Global Research Technologies, Novo Nordisk A/S, Novo Nordisk Park, DK-2760 Måløv, Denmark; 4 Institut de Chimie de Strasbourg, UMR 7177, CNRS, Université de Strasbourg, 1 rue Blaise Pascal, 67008 Strasbourg Cedex, France

Dear Sir,

Following the publication of our article entitled “Direct comparison of the in vitro and in vivo stability of DFO, DFO* and DFOcyclo* for ^89^Zr-immunoPET” in EJNMMI [1], readers sent us some remarks and questions. In this letter, we would like to address these questions by providing additional information about our study.

- DFOcyclo* is derived from DFO and not from DFO*. In DFO*, the trihydroxamate DFO is elongated with an extra hydroxamate group, exactly the same as present in DFO. In DFOcyclo*, the DFO is elongated with a cyclic hydroxamate group having a different linker length.
- During the manuscript review process, DFO*-NCS was made commercially available at ABX (catalogue number 7272). Therefore, the following statement in the introduction “However, in the absence of an improved, clinically applicable chelator, there is room for more efficient [^89^Zr]Zr^4+^ chelators” is no more correct.
- The stability assays were performed in PBS at pH 7.4.
- The amounts of DFO and EDTA used for the challenge experiments were calculated based on the amount of chelators.
- DFOcyclo* was used as a racemate in the experiments reported in the article.
- The results depicted in Figs. 4c and 5a are based on two different sets of experiments. Initially the in vivo stability of [^89^Zr]Zr-DFOcyclo*-trastuzumab was compared with [^89^Zr]Zr-DFO-trastuzumab, of which the results are depicted in Fig. 4c. Once we observed an improved in vivo stability of [^89^Zr]Zr-DFOcyclo*-trastuzumab, a subsequent in vivo study was performed to investigate its stability compared with [^89^Zr]Zr-DFO*-trastuzumab as well (Fig. 5). Although aimed at similar experimental conditions, the use of a freshly thawed cell line and new mice could have caused heterogeneity between the two experiments (e.g., increased tumor growth rates which result in different interstitial pressures that could affect the %ID/g), which could explain the slight differences in tumor uptake, blood kinetics, and uptake in other organs.
